# Biperiodic superlattices and transparent states in graphene

**DOI:** 10.1038/s41598-021-04690-x

**Published:** 2022-01-17

**Authors:** J. J. Alvarado-Goytia, R. Rodríguez-González, J. C. Martínez-Orozco, I. Rodríguez-Vargas

**Affiliations:** 1 Unidad Académica de Física, Universidad Autónoma de Zacatecas, Calzada Solidaridad Esquina con Paseo La Bufa S/N, 98060 Zacatecas, ZAC Mexico; 2 Unidad Académica de Ciencia y Tecnología de la Luz y la Materia, Universidad Autónoma de Zacatecas, Carretera Zacatecas-Guadalajara Km. 6, Ejido La Escondida, 98160 Zacatecas, ZAC Mexico

**Keywords:** Electronic properties and materials, Electronic properties and materials

## Abstract

The transmission and transport properties of biperiodic graphene superlattices are studied theoretically. Special attention is paid to the so-called transparent states of biperiodic superlattices. A Dirac-like Hamiltonian is used to describe the charge carriers in graphene. The transfer matrix method and the Landauer–Büttiker formalism are implemented to obtain the transmittance and conductance, respectively. Similar results to those reported for Schrödinger electrons are obtained. However, in the case of Dirac electrons the splitted bands and the transparent states associated to the biperiodicity depend strongly on the angle of incidence as well as the character of the charge carriers. In fact, the dynamic of the splitted bands and transparent states is inverted for holes. The origin of transparent states is unveiled by obtaining an analytic expression for the transmittance. It is found that resonant transmission through single and double barriers gives rise to transparent states. Regarding the transport properties, it is possible to identify the fundamental changes caused by the biperiodicity. In particular, it is found a splitting, shifting, and diminishment of the conductance peaks with respect to the case of regular periodicity. This opens the door to corroborate experimentally the fundamental characteristics of biperiodic gated graphene superlattices through transport measurements.

## Introduction

Semiconductor superlattices have been fundamental to demonstrate quantum size effects in artificial structures^1,2^. Moreover, the plethora of phenomena intrinsic to these structures are the basis of multiple device applications, with quantum cascade lasers being a well-known example^3^. The vast majority of works are devoted to single-period semiconductor superlattices. However, biperiodic or double period semiconductor superlattices have some characteristics that are interesting from both the fundamental and technological standpoint. In particular, the minibands are splitted into two subminibands, one of the subminibands presents narrow energy resonances and the other broad energy ones. In addition, a special energy resonance named transparent state arises at the edge of one of the subminibands. These characteristics were experimentally verified by Coquelin et al.^4,5^ in finite biperiodic GaAs/AlGaAs superlattices using hot electron spectroscopy. Later, Sprung et al.^6^ studied theoretically the origin of transparent states in biperiodic superlattices. They found that the Bragg resonance turns into a transparent state located close to the band edge of the low (high) energy subminiband when the first (second) well is wider. Furthermore, the transparent state occurs at a fixed energy, regardless of the number of unit-cells in the superlattice.

In the case of graphene, a periodic potential gives rise to extra Dirac points in the band structure and a highly anisotropic propagation of the charge carriers^7,8^. Extra Dirac points arise once the periodic electrostatic potential surpasses a critical value. The extra Dirac points are located at the Fermi energy for the case of equal barrier-well widths, and present an energy shift for unequal barrier-well widths. Furthermore, the group velocity of the charge carriers in the extra Dirac points and even the original one is renormalized, becoming in extreme cases zero in one direction and unchanged in another. The extra Dirac points have been experimentally confirmed in lateral and moiré graphene superlattices^9,10^. This transition from isotropic to anisotropic properties is not exclusive of the periodic modulation. For instance, graphene nanoribbons with different edge orientations exhibit edge-dependent electronic and optical properties^11–14^. Here, it is also important to mention that there are recent breakthroughs in the fabrication of the so-called gated (electrostatic) graphene superlattices (GGSLs)^15,16^. One of the most attractive aspects of this type of superlattice is the tunability that can be achieved through electrostatic gating in contrast to moiré graphene superlattices. As in the case of semiconductor superlattices, most of the works in graphene superlattices are devoted to the study of single-period structures. In fact, there are extensive studies in electrostatic^17–20,20–23^, magnetic^24–29^, and strain^30–34^ graphene superlattices. Regarding biperiodic superlattices in graphene the few works found in the literature address aspects related to the electron transport, band structure, and resonant peak splitting^35–38^. For instance, Huo et al.^35^ investigated the transmission properties of biperiodic magnetic superlattices with asymmetric barriers, finding superior wave vector filtering characteristics of biperiodic magnetic superlattices over single periodic ones. The same authors^36^ studied the transport properties of asymmetric biperiodic magnetic graphene superlattices for parallel and antiparallel magnetic configurations. They found a giant magnetoresistance effect with a strong dependence on the asymmetry and interval of the magnetic barriers. The superior wave filtering characteristics and the giant magnetoresistance effect of biperiodic magnetic graphene superlattices are attractive for electron wave filters and magnetic reading devices, respectively. Tashima et al.^37^ studied the generation of new Dirac cones in graphene under double-periodic potentials. They found that the Dirac cones are generated sporadically following the Diophantine equation, in contrast to the consecutive appearance of the Dirac cones in single-periodic potentials. They also found that the energy cutoff of the linear dispersion relation in graphene is directly implicated in the generation of the sporadic Dirac cones. Xu et al.^38^ investigate the resonant peak splitting in finite biperiodic magnetic graphene superlattices. General expressions for the transmission probability and the resonant peaks were derived. They also found resonant peaks splitting induced by the periodicity and a resonant peak related to the unit-cell of two barriers and two wells. The unit-cell related peak unchanged as the period varies and drops quickly as the unit-cell asymmetry increases. The splitting characteristics are also confirmed in the conductance and shot-noise. As it is documented there is some progress in the understanding of the resonant peaks splitting in biperiodic magnetic graphene superlattices. However, we consider that transparent states have not been studied in detail in biperiodic GGSLs (BPGGSLs). Specifically, the role played by the angle of incidence, the character of the charge carriers (electrons–holes), and the resonant characteristics within the unit-cell. Taking into account the relevance of BPGGSLs from both the fundamental and technological standpoint, we consider that a thorough assessment of its characteristics, including the transparent states, is necessary.

In this paper, we address biperiodic superlattices and transparent states in graphene. We first show the general characteristics of biperiodic superlattices and transparent states for Dirac electrons, highlighting the fundamental differences with respect to Schrödinger electrons. We then proceed to analyze the origin of the transparent states. Our analysis is based on an analytic expression for the transmission coefficient. Finally, we assess the impact of the biperiodic modulation on the transport properties. In particular, we analyze the linear-regime conductance at zero temperature varying the degree of biperiodicity as well as correlating it with the contour maps of the transmission.

## Theoretical model

**Figure 1:**
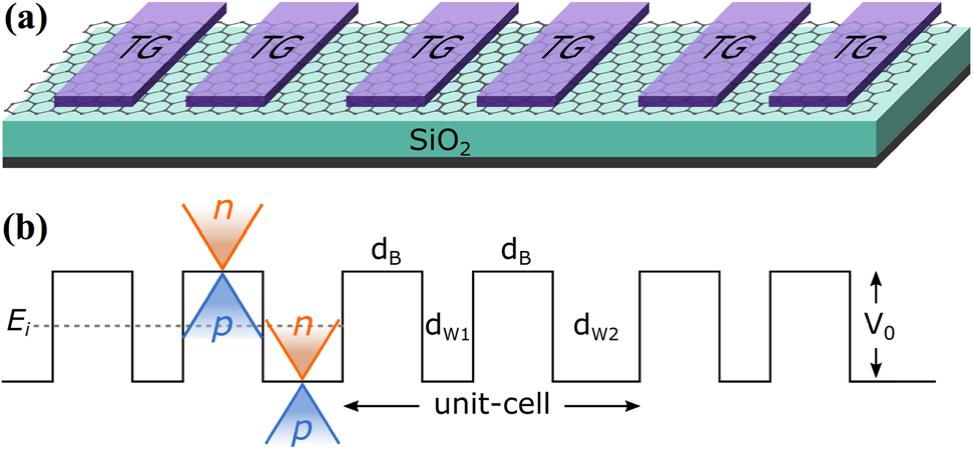
(**a**) Schematic representation of biperiodic graphene superlattices. Monolayer graphene is placed on a supporting substrate such SiO$_2$ and nanostructured with metallic electrodes or top gates (TGs) in biperiodic fashion. (**b**) Biperiodic potential profile along the superlattice axis. The superlattice unit-cell is composed of two metallic electrodes (barriers) and two inequivalent free regions (wells). $V_0$ and $d_B$ represent the height and width of barriers, while $d_{W1}$ and $d_{W2}$ the width of the first and second well in the superlattice unit-cell, respectively. Here, the number of superlattice periods is $N=3$. The *n* and *p* type regions in the superlattice structure are also highlighted. Depending on the energy of the incident charge carriers $E_i$, it is possible to have three different transport regions: for $E_i > V_0$ transport mediated exclusively by electrons, for $0<E_i<V_0$ the transport is owing to electrons and holes, and $E_i<0$ the transport is mediated only by holes.

In Fig. 1a we show a schematic representation of BPGGSLs. It consists of monolayer graphene placed on a supporting substrate such SiO$_2$ and top gates (TGs) arranged in biperiodic fashion. Two TGs alternated with two free regions constitute the unit-cell of BPGGSLs. The biperiodic potential profile is shown in Fig. 1(b). As can be noticed the two barriers in the unit-cell have the same height $V_0$ and the same width $d_B$, while the wells have dissimilar widths $d_{W1}$ and $d_{W2}$. The *n* and *p* type regions in the superlattice structure are also highlighted. Depending on the energy of the incident charge carriers $E_i$, it is possible to have transport mediated exclusively by electrons ($E_i>V_0$), electrons and holes ($0<E_i<V_0$), and exclusively by holes ($E_i<0$).

The charge carriers in BPGGSLs can be described by the low-energy effective Hamiltonian

1 $$\begin{aligned} {\hat{H}}= v_F \vec{ \sigma } \cdot \vec{p} + V(x), \end{aligned}$$

where

2 $$\begin{aligned} V(x)=\left\{ \begin{array}{@{\quad }l@{\quad }l@{}} V_0 &{} \text {for barriers} \\ 0 &{} \text {for wells} \end{array} \right. \end{aligned}$$

Here, $v_F$ is the Fermi velocity, $\vec{p}=(p_x,p_y)$ is the two-dimensional momentum and $\vec{\sigma }=(\sigma _x,\sigma _y)$ is the vector of Pauli matrices related to the sublattice pseudospin.

The wave function and wave vector in the barriers are given as

3 $$\begin{aligned} \psi ^b(x,y)= A^b_{+} \left( \begin{array}{c} 1 \\ v_{+} \end{array} \right) e^{ i q_x x + i q_y y } +A^b_{-} \left( \begin{array}{c} 1 \\ v_{-} \end{array} \right) e^{- i q_x x + i q_y y }, \end{aligned}$$

where

4 $$\begin{aligned} v_{\pm }=\frac{\hbar v_F \left( \pm q_x + i q_y \right) }{E-V_0} \end{aligned}$$

and

5 $$\begin{aligned} q_x= \frac{1}{\hbar v_F} \sqrt{ \left( E - V_0 \right) ^2- \hbar ^2 v^2_F q^2_y}. \end{aligned}$$

In the case of the wells and the left and right semi-infinite regions the wave function and wave vector are given in similar fashion. Actually, we can obtain them by simply setting $V_0=0$. In this case, we will use *W* as superscript, $u_{\pm }$ as bispinor coefficients and $\vec{k}=(k_x,k_y)$ as two-dimensional wave vector.

The transmission properties can be obtained with the help of the transfer matrix method. In fact, by requiring the continuity of the wave function along the superlattice structure as well as the conservation of the transverse wave vector $k_y=q_y$, we can relate the wave function coefficients of the left semi-infinite region $A^L_{+}$ and $A^L_{-}$ with the corresponding ones to the right semi-infinite region $A^R_{+}$ and $A^R_{-}$ through the so-called transfer matrix

6 $$\begin{aligned} \left( \begin{array}{c} A^L_+ \\ A^L_- \end{array} \right) = M^{BSL} \left( \begin{array}{c} A^R_+ \\ A^R_- \end{array} \right) \end{aligned}$$

where

7 $$\begin{aligned} M^{BSL}=\left[ M^{uc} \right] ^N, \end{aligned}$$

$M^{uc}$ being the transfer matrix of the superlattice unit-cell. With the help of Eq. (6) the transmission probability or transmittance can be written as

8 $$\begin{aligned} T=\left| \frac{A^R_+}{A^L_+} \right| ^2=\frac{1}{\left| M^{BSL}_{11} \right| ^2}, \end{aligned}$$

with $M^{BSL}_{11}$ the (1,1) element of $M^{BSL}$. By using the relationships between the transfer matrix elements^41^ we can write the transmittance as

9 $$\begin{aligned} T=\frac{1}{1+\left| M^{BSL}_{12} \right| ^2}, \end{aligned}$$

where $M^{BSL}_{12}$ is the (1,2) element of $M^{BSL}$. Now, by considering the Chebyshev’s identity^39,40^

10 $$\begin{aligned} \left[ M^{uc} \right] ^N= \left( \begin{array}{cc} M^{uc}_{11} &{} M^{uc}_{12} \\ M^{uc}_{21} &{} M^{uc}_{22} \end{array} \right) ^N= \left( \begin{array}{cc} M^{uc}_{11} U_{N-1} - U_{N-2} &{} M^{uc}_{12} U_{N-1} \\ M^{uc}_{21} U_{N-1} &{} M^{uc}_{22} U_{N-1} - U_{N-2} \end{array} \right) \end{aligned}$$

where

11 $$\begin{aligned} U_{N}=\frac{\sin \left( (N+1) q_{BL} d_{BL} \right) }{ \sin \left( q_{BL} d_{BL} \right) }, \end{aligned}$$

it is possible to write the transmittance in the form

12 $$\begin{aligned} T=\frac{1}{1+\left| M^{uc}_{12} \right| ^2 \frac{\sin ^2 \left( N q_{BL} d_{BL} \right) }{ \sin ^2 \left( q_{BL} d_{BL} \right) }}. \end{aligned}$$

Here, $q_{BL}$ and $d_{BL}$ are the Bloch wave vector and the size of the superlattice unit-cell, respectively. $q_{BL}$ is given by the trace of $M^{uc}$,

13 $$\begin{aligned} \cos \left( q_{BL} d_{BL} \right) = \frac{1}{2} \text{ Tr } \left[ M^{uc} \right] . \end{aligned}$$

We can obtain a more elaborated expression for the transmittance by developing explicitly $M^{uc}$. In this regard, $M^{uc}$ can be written as

14 $$\begin{aligned} M^{uc}=M_B M_{W1} M_B M_{W2}, \end{aligned}$$

where $M_B$, $M_{W1}$ and $M_{W2}$ are the transfer matrices of the barriers, first and second well of the superlattice unit-cell, respectively. These matrices are given by

15 $$\begin{aligned} M_{B}= & {} D^{-1}_0 (D_B P_B D^{-1}_B) D_0, \end{aligned}$$

16 $$\begin{aligned} M_{W1}= & {} P_{W1}, \end{aligned}$$

17 $$\begin{aligned} M_{W2}= & {} P_{W2}, \end{aligned}$$

where

18 $$\begin{aligned} D_B= & {} \left( \begin{array}{cc} 1 &{} 1 \\ v_+ &{} v_- \end{array} \right) , \nonumber \\ D_W= & {} \left( \begin{array}{cc} 1 &{} 1 \\ u_+ &{} u_- \end{array} \right) \end{aligned}$$

and

19 $$\begin{aligned} P_B= & {} \left( \begin{array}{cc} e^{-iq_x d_B } &{} 0 \\ 0 &{} e^{iq_x d_B} \end{array} \right) , \nonumber \\ P_{W1}= & {} \left( \begin{array}{cc} e^{-ik_x d_{W1} } &{} 0 \\ 0 &{} e^{ik_x d_{W1}} \end{array} \right) , \nonumber \\ P_{W2}= & {} \left( \begin{array}{cc} e^{-ik_x d_{W2} } &{} 0 \\ 0 &{} e^{ik_x d_{W2}} \end{array} \right) \end{aligned}$$

are the dynamic and propagation matrices of the barrier and well regions of the superlattice unit-cell. After some algebra, see Appendix A in the Supplementary Information, we arrive to a more elaborate expression for the transmittance:

20 $$\begin{aligned} T=\frac{1}{1+\left| M^{1B}_{12} \right| ^2 \left[ \text{ Tr } \left( M_B M_{W1} \right) \right] ^2 \frac{\sin ^2 \left( N q_{BL} d_{BL} \right) }{ \sin ^2 \left( q_{BL} d_{BL} \right) }}, \end{aligned}$$

where $M^{1B}_{12}$ is the (1,2) element of $M_B$, given by

21 $$\begin{aligned} M^{1B}_{12}=-\frac{k_y \left( k_x - i k_y \right) }{k_x q_x} \left( 1 - \frac{ s_q |q| }{ s_k |k|} \right) \sin \left( q_x d_{B} \right) \end{aligned}$$

and $\text{ Tr } \left( M_B M_{W1} \right)$ the trace of the transfer matrix of the first barrier and well of the superlattice unit-cell, namely:

22 $$\begin{aligned} \text{ Tr } \left( M_B M_{W1} \right) = 2 \left\{ \cos (q_x d_B) \cos (k_x d_{W1} ) + \frac{ \left( k^2_y - s_k s_q |k| |q| \right) }{k_x q_x} \sin (q_x d_B) \sin (k_x d_{W1}) \right\} . \end{aligned}$$

Here, $s_q=\text{ sgn }(E-V_0)$ and $s_k=\text{ sgn } (E)$ are the energy-dependent sign functions of the barriers and wells, respectively.

Likewise, the trace of $M^{uc}$ can be written as

23 $$\begin{aligned} \text{ Tr } \left[ M^{uc} \right] = \text{ Tr } \left( M_B M_{W1} \right) \text{ Tr } \left( M_B M_{W2} \right) - 2 \cos \left( k_x \left( d_{W1} - d_{W2} \right) \right) , \end{aligned}$$

where $\text{ Tr } \left( M_B M_{W2} \right)$ is given in similar fashion as Eq. (22), but what enters in the expression is $d_{W2}$ instead of $d_{W1}$. The details of this derivation for the $\text{ Tr } \left[ M^{uc} \right]$ can be found in the Appendix A of the Supplementary Information.

This set of expressions allows us to compute the transmittance of BPGGSLs. More importantly, they give us the possibility to know the origin of the different resonances in the transmittance.

The transport calculations are based on the Landauer–Büttiker formalism. In particular, the linear-regime conductance at zero temperature is computed with the formula

24 $$\begin{aligned} G(E_F)=G_0 \int ^{\pi /2}_{-\pi /2} T(E_F,\theta ) \cos \theta d \theta , \end{aligned}$$

where $G_0=2e^2L_y E_F/ h^2 v_F$ is the fundamental conductance factor, with $L_y$ the width of the graphene sheet and $E_F$ the Fermi energy of the charge carriers.

## Results and discussion

Here, we will show firstly the general characteristics of the transmission properties of BPGGSLs, paying special attention to the dependence of transparent states on the angle of incidence, the width of the quantum wells, and the character of the charge carriers. Then, we will proceed to analyze the origin of transparent states based on an analytic expression for the transmission coefficient. In addition, we will analyze the band structure and group velocity characteristics of transparent states in single and double periodic GGSLs. Finally, we will address the transport properties of BPGGSLs. Specifically, we will analyze the impact of the biperiodicity on the linear-regime conductance at zero temperature. It is also important to mention that throughout the study the height of the barriers will be $V_0=0.1$ eV. For this value of the potential there is no room for extra Dirac points^8,37^. So, the formation of extra Dirac points is not relevant for our analysis.

### General transmission characteristics of BPGGSLs

**Figure 2:**
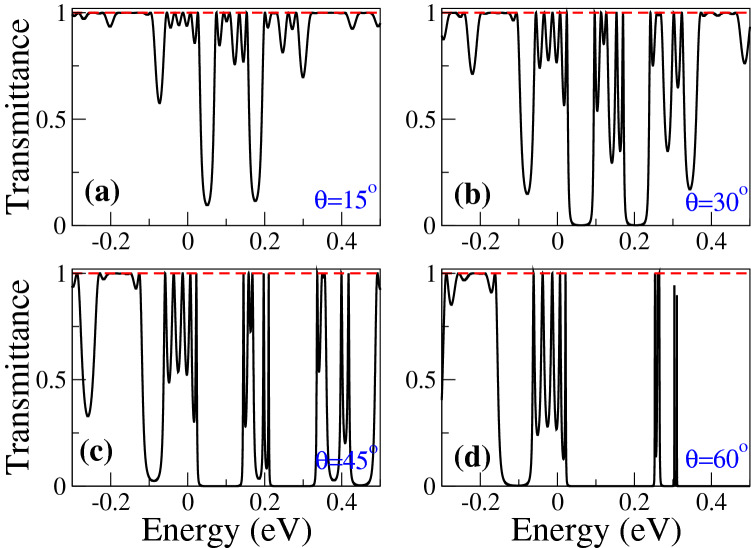
Transmittance as a function of the energy for BPGGSLs with $d_{W1} > d_{W2}$. Different incident angles have been considered: (**a**) $\theta =15^{\circ }$, (**b**) $\theta =30^{\circ }$, (**c**) $\theta =45^{\circ }$ and (**d**) $\theta =60^{\circ }$. In all cases the perfect transmission at normal incidence $\theta =0^{\circ }$ (dashed-red curve) is a manifestation of the Klein tunneling. Here, the superlattice parameters are: $V_0=0.1$ eV, $d_B=50a$, $d_{W1}=60a$, $d_{W2}=50a$ and $N=3$.

**Figure 3:**
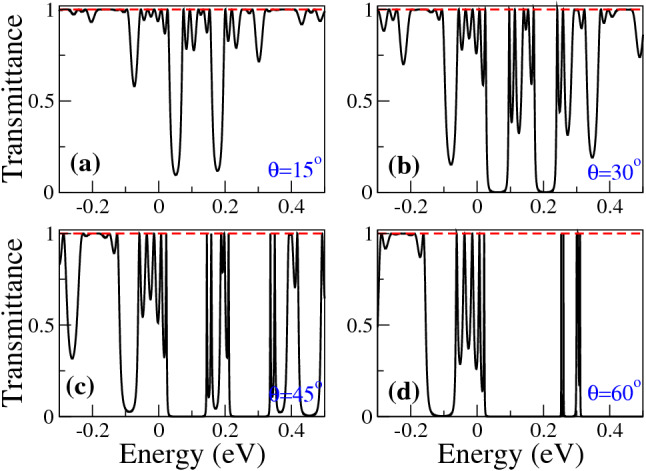
The same as Fig. 2, but for BPGGSLs with $d_{W1} < d_{W2}$. In particular, $d_{W1}=50a$ and $d_{W2}=60a$.

**Figure 4:**
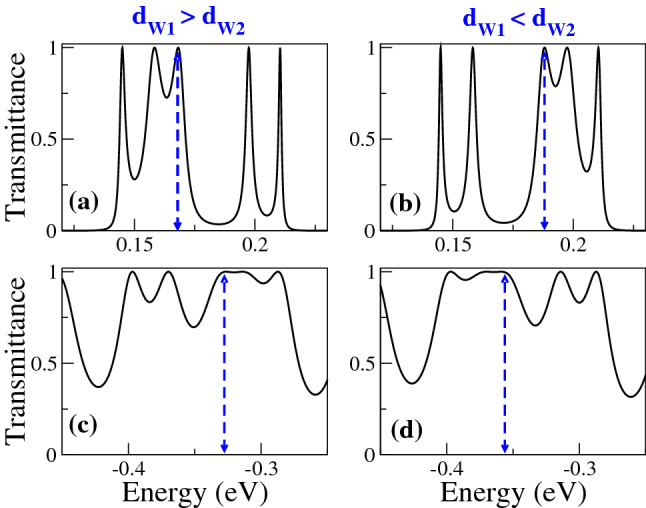
Transmission bands of BPGGSLs in which transparent states are clearly manifested. (**a**) and (**b**) correspond to electron transmission bands, while (**c**) and (**d**) to hole transmission bands. The resonance associated to the transparent state is indicated with the dashed-blue vertical arrow. The angle of incidence in all cases is $\theta =45^{\circ }$. The superlattice parameters of (**a**) and (**c**) are the same as in Fig. 2 and the ones of (**b**) and (**d**) are the same as in Fig. 3.

**Figure 5:**
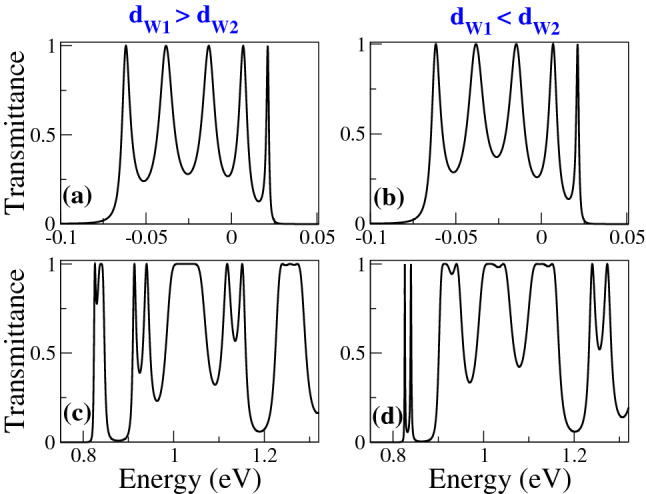
Transmission bands of BPGGSLs in which is not evident the contribution of transparent states. The angle of incidence in all cases is $\theta =60^{\circ }$. The superlattice parameters of (**a**) and (**c**) are the same as in Fig. 2 and the ones of (**b**) and (**d**) are the same as in Fig. 3.

In Fig. 2, we show the transmittance of BPGGSLs when $d_{W1}>d_{W2}$ for different angles of incidence: (a) $\theta =15^{\circ }$, (b) $\theta =30^{\circ }$, (c) $\theta =45^{\circ }$ and (d) $\theta =60^{\circ }$. The dashed-red lines correspond to normal incidence $\theta =0^{\circ }$, manifesting the well-known Klein tunneling of gated graphene structures^42^. As we can notice the minibands and minigaps are better defined as the angle of incidence increases. Moreover, the minigaps get larger and the minibands tend to degenerate as the angle increases. We can also see the splitting of the minibands as a consequence of the biperiodicity, however, the splitting is not the same for all minibands as in the case of Schrödinger electrons^6^. Similar transmittance characteristics are obtained when $d_{W1}<d_{W2}$, as shown in Fig. 3. In Fig. 4 we show the splitting of some (first row) electron and (second row) hole minibands when (first column) $d_{W1}>d_{W2}$ and (second column) $d_{W1}<d_{W2}$. The angle of incidence considered is $\theta =45^{\circ }$, so the energy minibands are sufficiently defined such that we can identify the miniband splitting and the different resonances within the subminibands. In fact, the splitting of electron minibands is equivalent to the corresponding one of Schrödinger electrons, that is, the broad resonances are located in the low-energy (high-energy) subminiband when $d_{W1}>d_{W2}$ ($d_{W1}<d_{W2}$). In addition, the transparent state is located at the edge of the subminiband of broad peaks, see the vertical dashed-blue arrows in Fig. 4a, b. Regarding hole minibands, we can see that the splitting is not as marked as for electron minibands, due to the biperiodic potential is on the electron energy side $E>0$. The splitting is also reversed with respect to electron minibands, that is, the broad resonances, including the transparent state, are in the high-energy (low-energy) subminiband when $d_{W1}>d_{W2}$ ($d_{W1}<d_{W2}$), as shown in Fig. 4c, d. We can also notice that not all minibands follow the splitting dynamic abovementioned for electron and hole minibands. For instance, the miniband around $E=0$ does not present any splitting no matter if $d_{W1}>d_{W2}$ or $d_{W1}<d_{W2}$, as shown in Fig. 5a, b. Actually, it is like a miniband of single-period GGSLs (SPGGSLs), with the resonances almost equally spaced and in number proportional to the quantum wells in the structure. For other minibands the splitting dynamic is more intricate as in the case of electron minibands at high energies, see Fig. 5c, d. For these minibands, it is difficult to say that they split in two subminibands of narrow and broad peaks located energetically according to the relation between $d_{W1}$ and $d_{W2}$.

**Figure 6:**
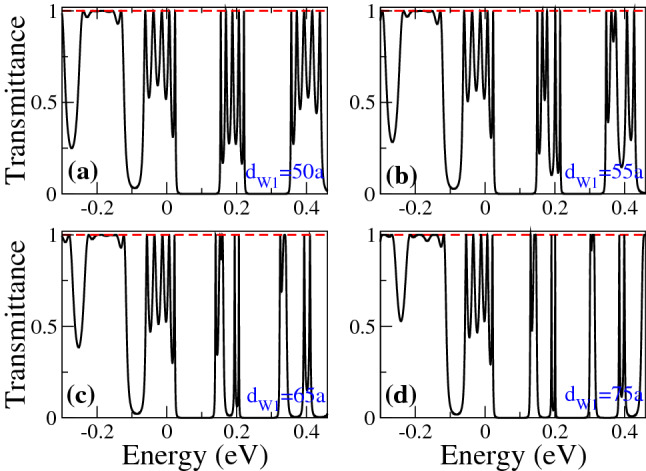
Transmittance of BPGGSLs as a function of the energy for different $d_{W1}$ as indicated. In all cases the other superlattices parameters are: $\theta =45^{\circ }$, $d_{W2}=50a$, $V_0=0.1$ eV and $N=3$.

**Figure 7:**
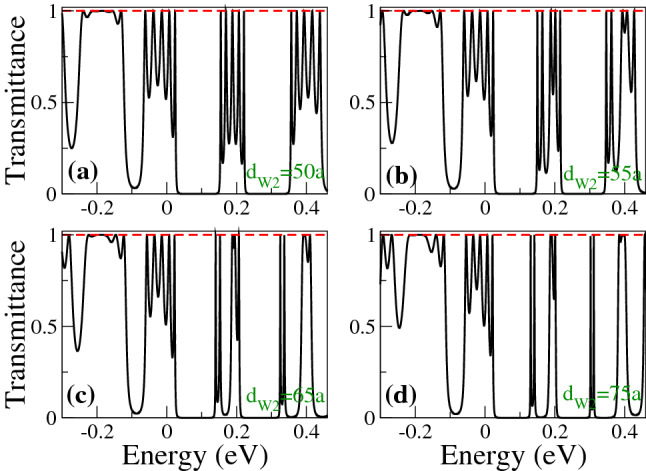
Transmittance of BPGGSLs as a function of the energy for different $d_{W1}$ as indicated. In all cases the other superlattices parameters are: $\theta =45^{\circ }$, $d_{W2}=50a$, $V_0=0.1$ eV and $N=3$.

**Figure 8:**
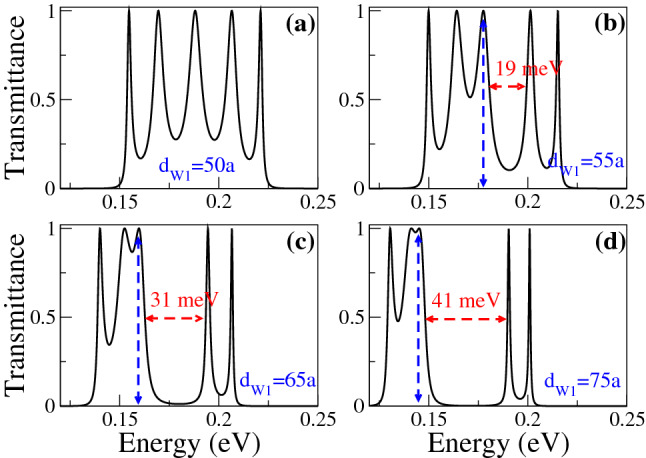
Main electron transmission band of BPGGSLs as a function of the energy for different $d_{W1}$ as indicated. The dashed-blue vertical arrows indicate the resonance associated to the transparent state. The other superlattice parameters are the same as in Fig. 6.

**Figure 9:**
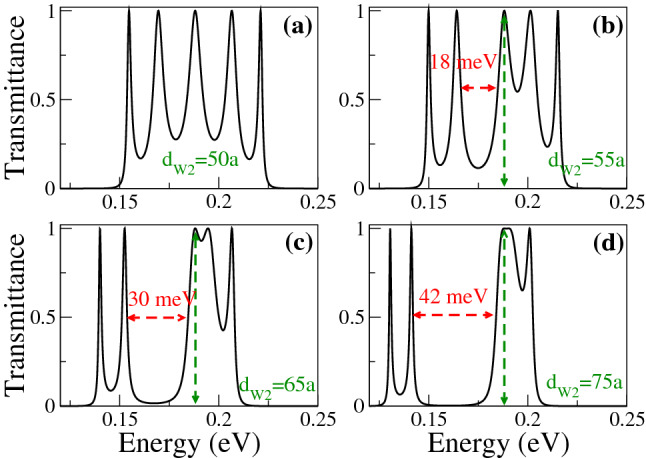
Main electron transmission band of BPGGSLs as a function of the energy for different $d_{W2}$ as indicated. The dashed-green vertical arrows indicate the resonance associated to the transparent state. The other superlattice parameters are the same as in Fig. 7.

In Fig. 6 we show the transmittance as a function of the energy for different $d_{W1}$ as indicated. The width of the second well is fixed at 50*a* and the angle of incidence considered is $\theta =45^{\circ }$. Figure 6a corresponds to SPGGSLs since $d_{W1}=50a$, consequently, the number of resonances within the energy minibands is proportional to the number of wells in the superlattice. Once biperiodicity is induced $d_{W1}=55a$ the energy minibands split, except the one around $E=0$, as shown in Fig. 6b. As $d_{W1}$ increases the subminigap between the subminibands increases, the subminibands shift to lower energies and the resonances get closer to each other, see Fig. 6c, d. A similar energy miniband dynamic is presented when we vary $d_{W2}$, keeping fixed $d_{W1}$, as shown in Fig. 7. The fundamental difference between Figs. 6 and 7 is the location of the narrow and broad resonances in the electron and hole subminibands. In Fig. 8 we focus on the evolution of the low-energy electron miniband as $d_{W1}$ varies. In particular, we find that the minigap gets larger as $d_{W1}$ increases, going from 0 to 41 meV as $d_{W1}$ increases from 50*a* to 75*a*. The subminibands also shift to lower energies, about 12 meV for the same variation of $d_{W1}$. Furthermore, the effective width of the miniband (subminibads+minigap) remains the same as $d_{W1}$ varies, about 70 meV. A similar evolution is obtained for the low-energy electron miniband as $d_{W2}$ varies, with the roles inverted between the narrow and broad resonance energy subminibands, as shown in Fig. 9.

### Origin of transparent states

**Figure 10:**
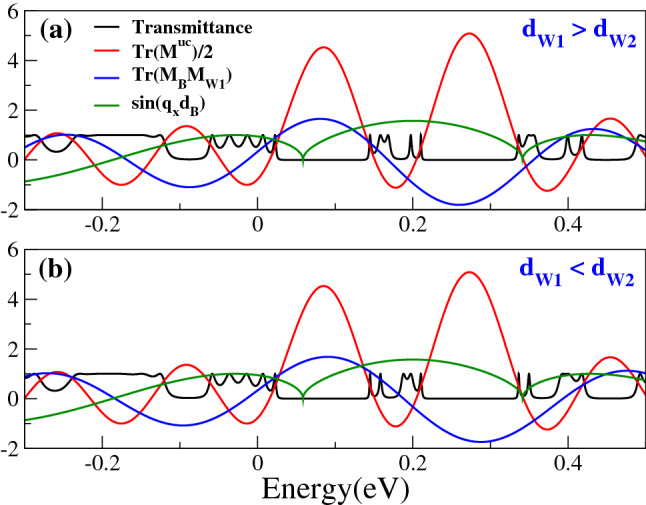
(**a**) Transmittance, trace of the biperiodic unit-cell $\text{ Tr }(M^{uc})/2$, trace of the first barrier and well $\text{ Tr }(M_B M_{W1})$ and $\sin (q_x d_B)$ as a function of the energy for BPGGSLs with $d_{W1} > d_{W2}$. The angle of incidence is $\theta =45^{\circ }$. The other superlattice parameters are the same as in Fig. 2. (**b**) The same as (**a**) but for $d_{W1} < d_{W2}$. The superlattice parameters are the same as in Fig. 3.

**Figure 11:**
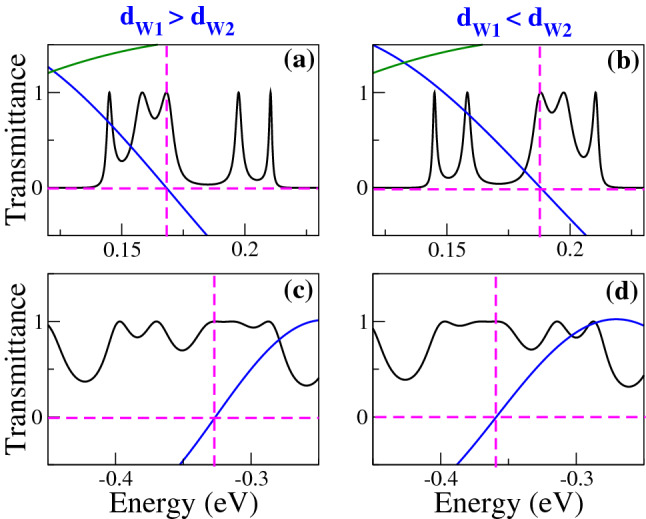
The same as Fig. 4, but here $\text{ Tr }(M_BM_{W1})$ (solid-blue curve) and $\sin (q_x d_B)$ (solid-dark-green curve) are included to unravel the origin of transparent states. The dashed-magenta lines help to identify the energies at which $\text{ Tr }(M_BM_{W1})$ and $\sin (q_x d_B)$ are zero.

**Figure 12:**
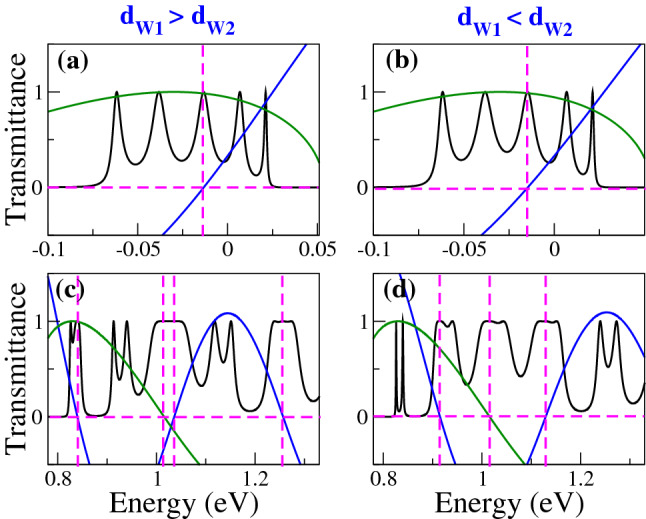
The same as Fig. 5, but here $\text{ Tr }(M_BM_{W1})$ (solid-blue curve) and $\sin (q_x d_B)$ (solid-dark-green curve) are included to unravel the origin of transparent states. The dashed-magenta lines help to identify the energies at which $\text{ Tr }(M_BM_{W1})$ and $\sin (q_x d_B)$ are zero.

Now, it is turn to unravel the origin of transparent states. In order to do so, we will analyze all ingredients involved in the determination of the transmittance, see Eqs. (13) and (20). Specifically, the trace of the first barrier and well $\text{ Tr }(M_BM_{W1})$, the resonant tunneling condition of the first barrier $\sin (q_xd_B)$, the trace of the biperiodic unit-cell $\text{ Tr }(M^{uc})$, and the transmittance itself. The dependence of all these quantities with respect to the energy is shown in Fig. 10. Half of the trace of the biperiodic unit-cell gives us the allowed and forbidden energy regions for the electron states, minibands and minigaps, respectively. In particular, the condition $\left| \text{ Tr }(M^{uc})/2 \right| <1$ gives us the allowed energy regions, see the solid-red lines in Fig. 10. We can notice that the resonant tunneling condition $\sin (q_xd_B)$ is not implicated in the low-energy electron and hole minibands. It is also important to mention that the resonances that arise from this condition are independent of the degree of biperiodicity, that is, they are independent of $d_{W1}$ and $d_{W2}$. Once we defined $q_x$ and $d_B$ these resonances are fixed, however, they are not arising at low-energy and consequently, they are not the reason for transparent states. On the contrary, the transmittance and $\text{ Tr }(M_BM_{W1})$ are quantities that depend on the degree of biperiodicity. In fact, as we described in the previous section the roles between the subminibands with narrow and broad resonances are inverted according to the proportion between $d_{W1}$ and $d_{W2}$. $\text{ Tr }(M_BM_{W1})$ depends directly on $d_{W1}$, so if the proportion between the widths of the wells changes $\text{ Tr }(M_BM_{W1})$ changes as well. These characteristics can be appreciated in the solid-black and solid-blue curves of Fig. 10a, b. More importantly, we can see that transparent states are located exactly at the energies at which $\text{ Tr }(M_BM_{W1})=0$. This correspondence between transparent states and $\text{ Tr }(M_BM_{W1})$ is better appreciated for different electron and hole minibands in Figs. 11 and 12. Even, the resonance at the middle of the apparent regular miniband around $E=0$ is related to the $\text{ Tr }(M_BM_{W1})$, as shown in Fig. 12a, b. In the case of electron minibands at high energies, the $\text{ Tr }(M_BM_{W1})$ contributes with two and three resonances and an additional resonance is related to $\sin (q_xd_B)$, see Fig. 12c, d. This is the reason why we see a more intricate dynamic for these minibands.

**Figure 13:**
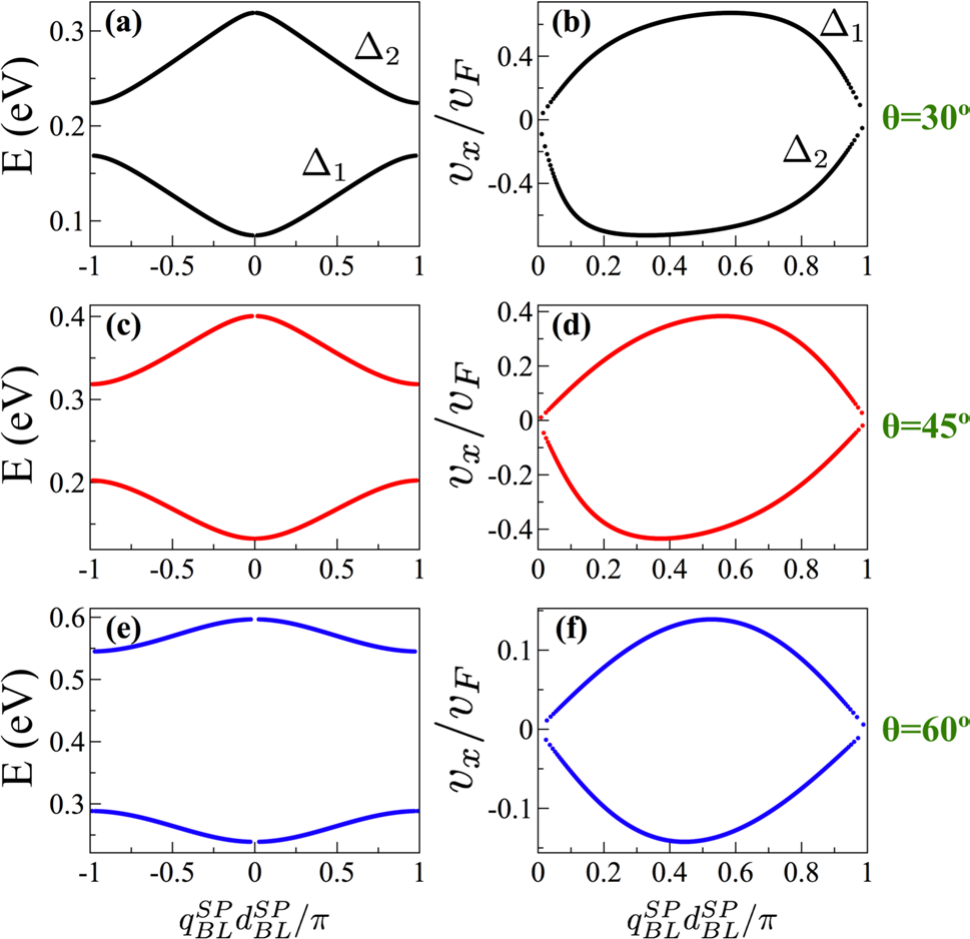
(First column) Low-energy dispersion relation and (Second column) group velocity $v_x$ of SPGGSLs for different angles of incidence as indicated. The single-period superlattice parameters are: $V_0=0.1$ eV, $d_B=50a$ and $d_{W1}=60a$. $\Delta _1$ and $\Delta _2$ represent the first and second electron energy minibands, respectively.

As we can realize there are several characteristics of biperiodic graphene superlattices that were not properly addressed by Xu et al.^38^. For instance, the role played by the charge carriers, the relevance of the angle of incidence, and the impact of the details of the biperiodic unit-cell. Regarding the latter, Xu et al.^38^ talk about unit-cell related peaks in generic terms, however, the resonant peaks associated to the unit-cell can be caused either by $\sin (q_xd_B)$ and/or $\text{ Tr }(M_BM_{W1})$ as we have documented earlier. In fact, we found that transparent states owe their origin to $\text{ Tr }(M_BM_{W1})=0$ as resonant tunneling condition. In the case of $\sin (q_xd_B)=0$, it is well-known that it represents the resonant tunneling condition of the barrier as resonant cavity. However, in the case of $\text{ Tr }(M_BM_{W1})=0$, it is not at all clear its physical meaning and its compatibility with the Sprung’s transparent state interpretation^6^. So, we proceed to analyze this condition in more detail. Actually, $\text{ Tr }(M_BM_{W1})$ is related directly to the band structure of SPGGSLs through the fundamental relation

25 $$\begin{aligned} 2\cos (q^{SP}_{BL}d^{SP}_{BL})=\text{ Tr }(M_BM_{W1}), \end{aligned}$$

where $q^{SP}_{BL}$ and $d^{SP}_{BL}$ are the Bloch wave vector and the size of the unit-cell of the single-period structure. In fact, the electron states of a single-period superlattice that fulfill with the condition for transparent states are those with a Bloch phase $\phi ^{SP}_{BL}=q^{SP}_{BL}d^{SP}_{BL}=\pm \pi /2$. In Fig. 13 we show the dispersion relation for (a) $\theta =30^{\circ }$, (c) $\theta =45^{\circ }$ and (e) $\theta =60^{\circ }$. Taking into account the form of the energy minibands, we can realize that the electron states at $\phi ^{SP}_{BL}=\pm \pi /2$ are electron states with high group velocity within the energy miniband. Remember that the group velocity can be computed through the derivative of the dispersion relation

26 $$\begin{aligned} v_x=\frac{1}{\hbar } \frac{\partial E(q^{SP}_{BL},\theta )}{\partial q^{SP}_{BL}}. \end{aligned}$$

The details of the expression for $v_x$ can be found in the Appendix B of the Supplementary Information. In Fig. 13b, d, f the group velocities corresponding to the dispersion relation of Fig. 13a, c, e are shown. As we can notice the electron states of the single-period structure that fulfill with the condition of transparent states have high group velocities within a specific energy miniband. We can also see that as the angle of incidence increases the mentioned states become states with maximum group velocity.

**Figure 14:**
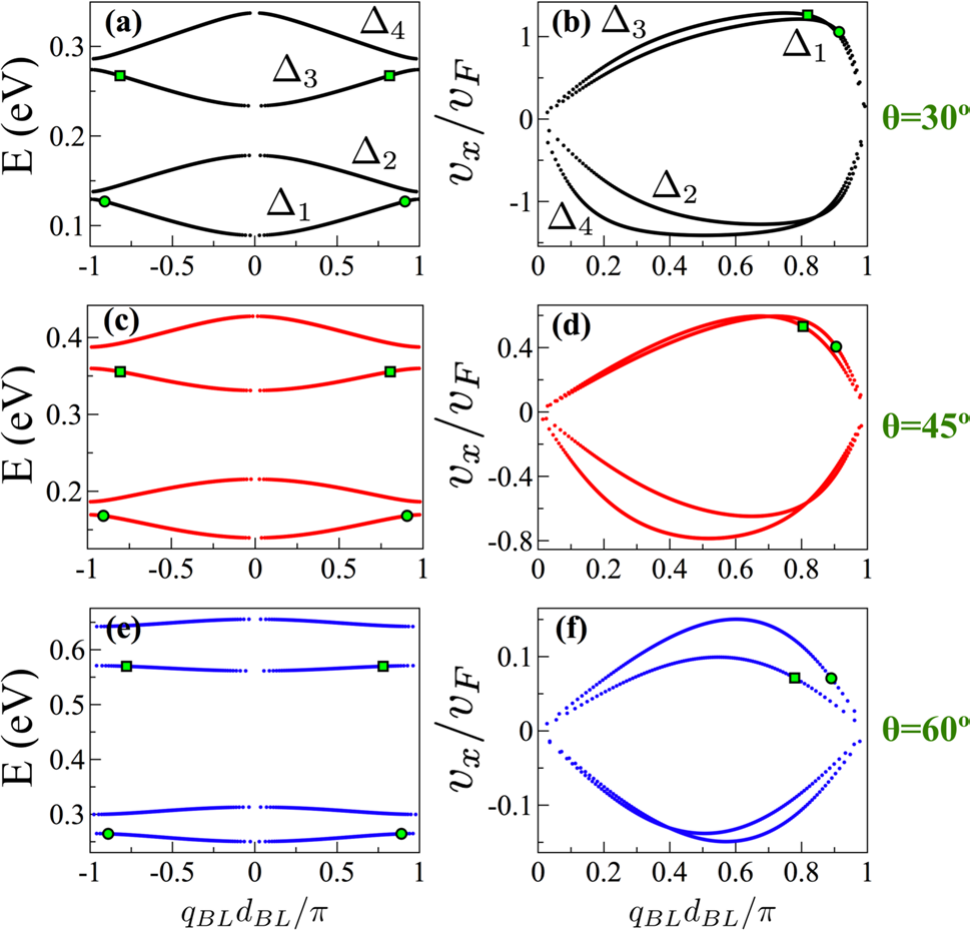
The same as Fig. 13, but for BPGGSLs. The biperiodic superlattice parameters are: $V_0=0.1$ eV, $d_B=d_{W2}=50a$ and $d_{W1}=60a$. $\Delta _1$, $\Delta _2$, $\Delta _3$ and $\Delta _4$ represent the first, second, third and fourth electron energy minibands, respectively. The points and squares in color correspond to transparent states.

However, when these states are in the biperiodic environment they are not necessarily states with high group velocity within electron energy minibands as shown in Fig. 14. The dispersion relation and the group velocity can be obtained in similar fashion as in the case of single-period superlattices. The details are presented in the Appendix B of the Supplementary Information. Actually, by considering the condition for transparent states $\text{ Tr }(M_BM_{W1})=0$ in the equation for the dispersion relation of BPGGSLs we can obtain

27 $$\begin{aligned} \cos (q_{BL}d_{BL})=-\cos (k_x(d_{W1}-d_{W2})), \end{aligned}$$

which solving for $q_{BL}$ yields

28 $$\begin{aligned} q_{BL}d_{BL}=k_x (d_{W1}-d_{W2})+\pi . \end{aligned}$$

Deriving this expression we can get readily the group velocity of transparent states

29 $$\begin{aligned} \frac{v^{ts}_x}{v_F}=\frac{d_{BL}}{d_{W1}-d_{W2}} \frac{k_x}{s_k|k|}. \end{aligned}$$

**Figure 15:**
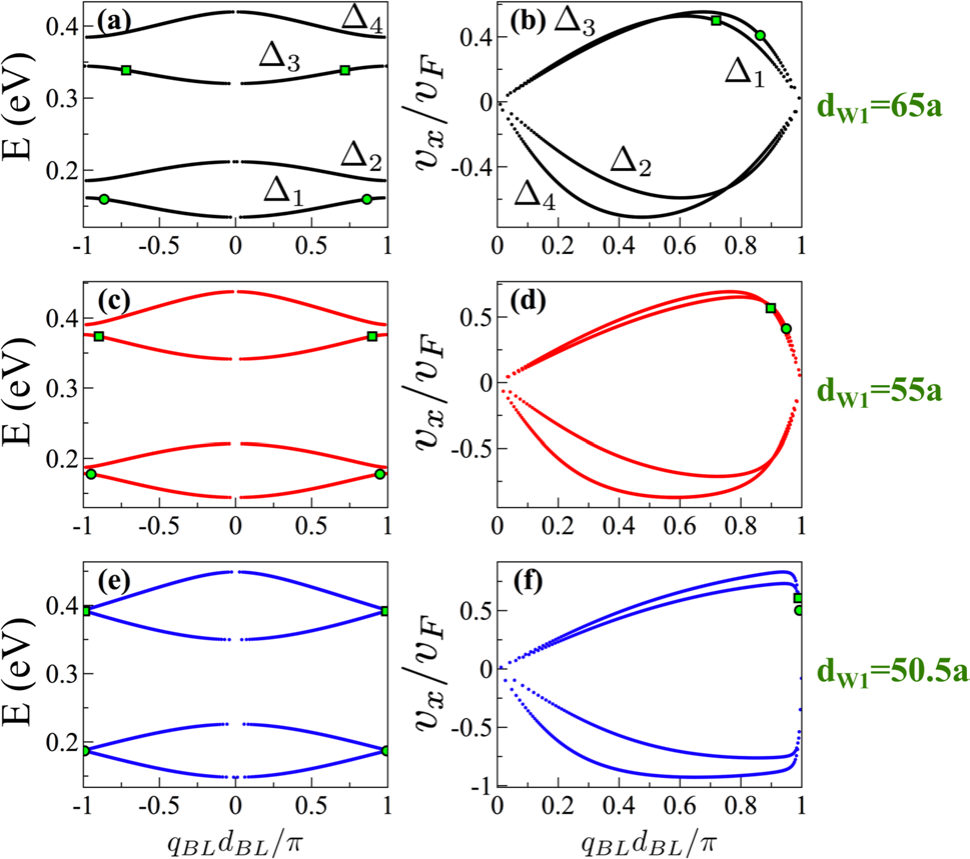
The same as Fig. 14, but here $d_{W1}$ is varied as indicated. The biperiodic superlattice parameters are: $V_0=0.1$ eV, $d_B=d_{W2}=50a$ and $\theta =45^{\circ }$. $\Delta _1$, $\Delta _2$, $\Delta _3$ and $\Delta _4$ represent the first, second, third and fourth electron energy minibands, respectively. The points and squares in color correspond to transparent states.

This expression tells us that as the difference between the widths of the wells, the degree of biperiodicity, is reduced $v^{ts}_x$ increases. Furthermore, as the degree of biperiodicity diminishes the transparent states are closer to the boundary of the biperiodic Brillouin zone. These characteristics can be appreciated in Fig. 15. In particular, see how the transparent states (points and squares in color) are moving as the degree of biperiodicity decreases. It is also important to mention that as $k_x/|k|=\cos \theta$, the angle of incidence is directly implicated in $v^{ts}_{x}$. In fact, as the angle of incidence increases the group velocity decreases. Finally, if we consider $d_{W1} < d_{W2}$ similar results are obtained, however the energy subminibands that harbor transparent states are the high energy ones, results not shown.

### Impact of biperiodicity on the transport properties

**Figure 16:**
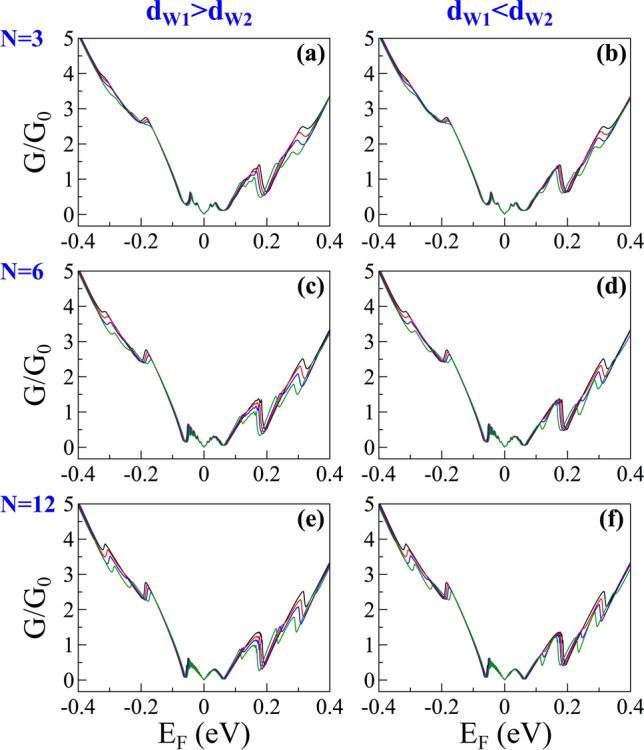
Conductance versus the Fermi energy of BPGGSLs for different number of periods *N* as indicated. In the left column ($d_{W1}>d_{W2}$) $d_{W2}$ is fixed to 50*a* and $d_{W1}$ takes values of 50*a*, 55*a*, 60*a* and 70*a*, black, red, blue and dark-green lines, respectively. In the right column ($d_{W1}<d_{W2}$) the roles between $d_{W1}$ and $d_{W2}$ are reversed. In all cases, the other superlattice parameters are: $d_B=50a$ and $V_0=0.13$ eV.

**Figure 17:**
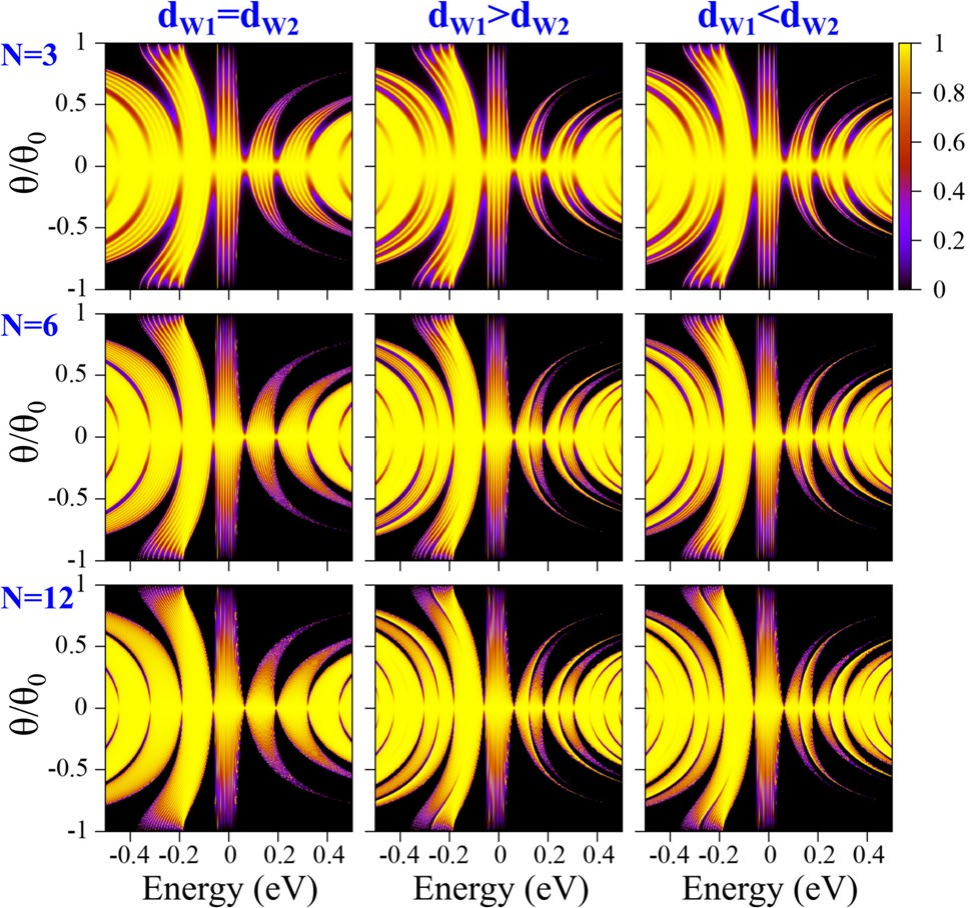
Transmission maps of BPGGSLs for different number of periods *N* as indicated. Here, the superlattice parameters are: (Left) $d_{W1}=d_{W2}=50a$, (Middle) $d_{W1}=60a$ and $d_{W2}=50a$, and (Right) $d_{W1}=50a$ and $d_{W2}=60a$. In all cases $d_B=50a$ and $V_0=0.13$ eV. Here, the angle of incidence is normalized to $\theta _0=90^{\circ }$.

Finally, we will analyze the impact of biperiodicity on the transport properties. Specifically, we want to see if an identifiable hallmark associated to the biperiodic potential is manifested in the zero temperature linear-regime conductance. In Fig. 16 we show the conductance outcomes for different degrees of biperiodicity when (first column) $d_{W1}>d_{W2}$ and (second column) $d_{W1}<d_{W2}$. We have considered different number of periods: (first row) $N=3$, (second row) $N=6$ and (third row) $N=12$. In all cases, the solid-black curve corresponds to the periodic case and serves as reference contrasting the fundamental changes related to the biperiodicty. As we can notice the conductance of the periodic case presents an oscillating ascending trend as the Fermi energy increases. This is a typical characteristic of periodic GGSLs, related to the formation of energy minibands, as shown in the first column of the transmission maps of Fig. 17. In fact, as the number of periods increases the minibands and minigaps are better defined and the resonances within the minibands increase as well. These characteristics give rise to steeper conductance curves as well as a peak structure within the main conductance peaks, see the second and third row in Fig. 16. Once the biperiodicity is induced the main conductance peaks shift to lower energies and reduce with respect to the periodic case, compare the solid-red and solid-black curves in Fig. 16. As the degree of biperiodicity increases the shifting and reduction of the conductance peaks gets larger, resulting in practically two peaks at 70*a* for either $d_{W1}>d_{W2}$ or $d_{W1}<d_{W2}$. We can also note that these changes are more notorious in the conductance curves that correspond to $d_{W1}>d_{W2}$. These differences are directly related to the splitting dynamics of the energy minibands of BPGGSLs as shown in the transmission maps for $d_{W1}>d_{W2}$ and $d_{W1}<d_{W2}$, second and third column of Fig. 17, respectively. In fact, when $d_{W1}>d_{W2}$ the low-energy subminibands cover a wider angular range, practically nesting the high-energy subminibands. This results in marked changes in the conductance since this quantity is the result of averaging the transmittance over all angles of incidence while keeping fixed the Fermi energy. In short, we have shown that biperiodicity effects can be identified on the transport properties, opening the door to corroborate the splitting of the energy minibands through transport measurements. Regarding transparent states, we cannot see their contribution directly on the transport properties. So, additional external effects such as magnetic field and/or strain effects are necessary in order to observe its impact directly on the transport and transport-related properties such as the conductance and the shot noise^38^.

## Conclusions

In summary, we have studied the transmission and transport properties of BPGGSLs. We paid special attention to the characteristics and origin of the transparent states. The transfer matrix method and the Landauer–Büttiker formalism were used to obtain the transmittance and the zero temperature linear-regime conductance, respectively. We found that once the biperiodicity is incorporated the superlattice transmission bands are splitted and transparent states arise in the edges of the splitted bands as in the case of Schrödinger electrons. However, the splitted bands and the transparent states of BPGGSLs depend strongly on the angle of incidence and the character of the charge carriers. More importantly, we obtained an analytic expression for the transmission coefficient that allows us to unravel the origin of transparent states. In fact, transparent states owe their origin to the resonant tunneling through single and double barriers. We also identify the fundamental changes caused by the biperiodicity and particularly by the transparent states on the band structure. In the case of the transport properties, we found that the splitting of the transmission bands results in additional peaks in the conductance and a diminution of it as the contrast between the width of the quantum wells increases, opening the door to corroborate experimentally the fundamental effects of BPGGSLs. Finally, we would like to remark that further studies with other external effects such as strain, interacting substrates, magnetic proximity effects, electromagnetic radiation, etc. and other 2D materials such as silicene, transition metal dichalcogenides, and phosphorene are needed in order to have a better understanding of biperiodic superlattices and transparent states for Dirac electrons.

## Supplementary Information

Supplementary Information.
