# Supplementary material for: Biperiodic superlattices and transparent states in graphene
Source: Sci Rep. 2022 Jan 17;12:832. doi: 10.1038/s41598-021-04690-x (PMC8764099; doi:10.1038/s41598-021-04690-x)
Supplement: Supplementary file 1 — Supplementary Information. [file 41598_2021_4690_MOESM1_ESM.pdf]

Supplementary Information

Biperiodic superlattices and transparent states in graphene

J. J. Alvarado-Goytia[1], R. Rodríguez-González[2], J. C. Martínez-Orozco[1] and I. Rodríguez-Vargas[2]

[1] *Unidad Académica de Física, Universidad Autónoma de Zacatecas, Calzada Solidaridad Esquina con Paseo La Bufa S/N, 98060 Zacatecas, Zac., Mexico.*

[2] *Unidad Académica de Ciencia y Tecnología de la Luz y la Materia, Universidad Autónoma de Zacatecas, Carretera Zacatecas-Guadalajara Km. 6, Ejido La Escondida, 98160 Zacatecas, Zac., Mexico.*

Appendix A: Biperiodic unit-cell transfer matrix

For completeness we will present explicitly the expressions for the different matrices involved in the computation of the transmittance and the trace of the biperiodic unit-cell. Let's start with the transfer matrix of the barriers $M_B = D_0^{-1}\left(D_B P_B D_B^{-1}\right)D_0$, where the triple product is given as

$$D_B P_B D_B^{-1} = \begin{pmatrix} \cos(q_x d_B) - \frac{k_y}{q_x}\sin(q_x d_B) & -\frac{i(E-V_0)}{\hbar v_F q_x}\sin(q_x d_B) \\ -\frac{i(E-V_0)}{\hbar v_F q_x}\sin(q_x d_B) & \cos(q_x d_B) + \frac{k_y}{q_x}\sin(q_x d_B) \end{pmatrix} \tag{A.1}$$

and multiplying by D_0^{-1} and D_0 we get

$$M_B = \begin{pmatrix} \cos(q_x d_B) + i\frac{(k_y^2 - s_k|k|s_q|q|)}{k_x q_x}\sin(q_x d_B) & -\frac{k_y(k_x - ik_y)}{k_x q_x}\left(1 - \frac{s_q|q|}{s_k|k|}\right)\sin(q_x d_B) \\ -\frac{k_y(k_x + ik_y)}{k_x q_x}\left(1 - \frac{s_q|q|}{s_k|k|}\right)\sin(q_x d_B) & \cos(q_x d_B) - i\frac{(k_y^2 - s_k|k|s_q|q|)}{k_x q_x}\sin(q_x d_B) \end{pmatrix}. \tag{A.2}$$

Here, we can see that $M_{11}^{1B} = (M_{22}^{1B})^*$, $M_{12}^{1B} = (M_{21}^{1B})^*$ and $|M_{11}^{1B}|^2 = 1 + |M_{12}^{1B}|^2$. The matrix elements of M_B help us to write the transfer matrix of the biperiodic unit-cell $M^{uc} = M_B M_{W1} M_B M_{W2}$ as

$$M^{uc} = \begin{pmatrix} \{(M_{11}^{1B})^2 e^{-ik_x d_{W1}} + |M_{12}^{1B}|^2 e^{ik_x d_{W1}}\} e^{-ik_x d_{W2}} & 2\mathrm{Re}\left(M_{11}^{1B} e^{-ik_x d_{W1}}\right) M_{12}^{1B} e^{ik_x d_{W2}} \\ 2\mathrm{Re}\left(M_{11}^{1B} e^{-ik_x d_{W1}}\right) \left(M_{12}^{1B}\right)^* e^{-ik_x d_{W2}} & \{((M_{11}^{1B})^*)^2 e^{ik_x d_{W1}} + |M_{12}^{1B}|^2 e^{-ik_x d_{W1}}\} e^{ik_x d_{W2}} \end{pmatrix}. \tag{A.3}$$

As $2\mathrm{Re}\left(M_{11}^{1B} e^{-ik_x d_{W1}}\right) = \mathrm{Tr}(M_B M_{W1})$, M_{12}^{uc} adopts the form

$$M_{12}^{uc} = M_{12}^{1B} \mathrm{Tr}(M_B M_{W1}) e^{ik_x d_{W2}}. \tag{A.4}$$

In similar fashion the trace of M^{uc} is given by

$$\mathrm{Tr}(M^{uc}) = 2\mathrm{Re}\left(\{(M_{11}^{1B})^2 e^{-ik_x d_{W1}} + |M_{12}^{1B}|^2 e^{ik_x d_{W1}}\} e^{-ik_x d_{W2}}\right), \tag{A.5}$$

where by replacing $|M_{12}^{1B}|^2 = |M_{11}^{1B}|^2 - 1$ yields

$$\mathrm{Tr}(M^{uc}) = 2\mathrm{Re}\left(\mathrm{Tr}(M_B M_{W1}) M_{11}^{1B} e^{-ik_x d_{W2}} - e^{ik_x (d_{W1} - d_{W2})}\right) \tag{A.6}$$

and by taking the real part, we obtain:

$$\mathrm{Tr}(M^{uc}) = \mathrm{Tr}(M_B M_{W1}) \mathrm{Tr}(M_B M_{W2}) - 2\cos(k_x (d_{W1} - d_{W2})). \tag{A.7}$$

Finally, it is important to mention that M^{uc} fulfills with the fundamental relations between the transfer matrix elements: $M_{11}^{uc} = (M_{22}^{uc})^*$, $M_{12}^{uc} = (M_{21}^{uc})^*$ and $|M_{11}^{uc}|^2 = 1 + |M_{12}^{uc}|^2$.

Appendix B: Group velocity SPGGSLs and BPGGSLs

The group velocity of SPGGSLs can be obtained by deriving Eq. (25) with respect to the energy

$$-2d_{BL}^{SP}\sin(q_{BL}^{S^P}d_{BL}^{SP})\frac{\partial q_{BL}^{SP}}{\partial E}=\frac{\partial\left[\mathrm{Tr}(M_B M_{W1})\right]}{\partial E}. \tag{B.1}$$

The derivative of the trace can be written as

$$\frac{1}{2}\frac{\partial\left[\mathrm{Tr}(M_B M_{W1})\right]}{\partial E}=-\frac{1}{f_{BL}^{SP}} \tag{B.2}$$

where

$$\frac{1}{f_{BL}^{SP}}=d_B f_1+d_{W1}f_2-f_3 \tag{B.3}$$

with

$$f_1=\frac{\partial q_x}{\partial E}\left\{\sin(q_x d_B)\cos(k_x d_{W1})-\frac{\left(k_y^2-s_k s_q|k||q|\right)}{k_x q_x}\cos(q_x d_B)\sin(k_x d_{W1})\right\}, \tag{B.4}$$

$$f_2=\frac{\partial k_x}{\partial E}\left\{\cos(q_x d_B)\sin(k_x d_{W1})-\frac{\left(k_y^2-s_k s_q|k||q|\right)}{k_x q_x}\sin(q_x d_B)\cos(k_x d_{W1})\right\}, \tag{B.5}$$

$$f_3=\frac{\partial}{\partial E}\left\{\frac{\left(k_y^2-s_k s_q|k||q|\right)}{k_x q_x}\right\}\sin(q_x d_B)\sin k_x d_{W1}, \tag{B.6}$$

and

$$\frac{\partial q_x}{\partial E}=\frac{s_q|q|}{\hbar v_F q_x},\quad \frac{\partial k_x}{\partial E}=\frac{s_k|k|}{\hbar v_F k_x},$$

$$\frac{\partial}{\partial E}\left\{\frac{\left(k_y^2-s_k s_q|k||q|\right)}{k_x q_x}\right\}=-\frac{s_k|k|+s_q|q|}{\hbar v_F k_x q_x}-\frac{\left(k_y^2-s_k s_q|k||q|\right)}{\hbar v_F k_x q_x}\left[\frac{s_k|k|}{k_x^2}+\frac{s_q|q|}{q_x^2}\right].$$

Finally, the group velocity can be expressed as

$$v_x = \frac{1}{\hbar} d_{BL}^{SP} \sin(q_{BL}^{SP} d_{BL}^{SP}) f_{BL}^{SP}. \tag{B.7}$$

In similar fashion, we can obtain the group velocity for BPGGSLs by deriving Eq. (13) with respect to the energy

$$-2\sin(q_{BL} d_{BL}) d_{BL} \frac{\partial q_{BL}}{\partial E} = \frac{\partial \left[\mathrm{Tr}(M^{uc})\right]}{\partial E}. \tag{B.8}$$

Defining the derivative of the trace as

$$\frac{1}{2}\frac{\partial \left[\mathrm{Tr}(M^{uc})\right]}{\partial E} = -\frac{1}{f_{BL}}, \tag{B.9}$$

we arrive to a similar equation for the group velocity as in the case of SPGGSLs,

$$v_x = \frac{1}{\hbar} d_{BL} \sin(q_{BL} d_{BL}) f_{BL}. \tag{B.10}$$

In accordance with Eq. (23) f_{BL} can be written as

$$\frac{1}{f_{BL}} = \left\{ \frac{\mathrm{Tr}(M_B M_{W2})}{f_{BL}^{SP1}} + \frac{\mathrm{Tr}(M_B M_{W1})}{f_{BL}^{SP2}} \right\} - (d_{W1} - d_{W2}) \frac{s_k |k|}{\hbar v_F k_x} \sin(k_x (d_{W1} - d_{W2})). \tag{B.11}$$

Here, f_{BL}^{SP1} and f_{BL}^{SP2} are given in similar fashion as Eq. (B.3), but using d_{W1} and d_{W2}, respectively.
